# In vitro digestion and lactase treatment influence uptake of quercetin and quercetin glucoside by the Caco-2 cell monolayer

**DOI:** 10.1186/1475-2891-4-1

**Published:** 2005-01-11

**Authors:** Jeanelle Boyer, Dan Brown, Rui Hai Liu

**Affiliations:** 1 Department of Animal Science, Cornell University, Ithaca, New York 14853-7201, USA; 2 Institute of Comparative and Environmental Toxicology, Cornell University, Ithaca, New York 14853-7201, USA; 3 Department of Food Science, Stocking Hall, Cornell University, Ithaca, NY 14853-7201, USA

## Abstract

**Background:**

Quercetin and quercetin glycosides are widely consumed flavonoids found in many fruits and vegetables. These compounds have a wide range of potential health benefits, and understanding the bioavailability of flavonoids from foods is becoming increasingly important.

**Methods:**

This study combined an in vitro digestion, a lactase treatment and the Caco-2 cell model to examine quercetin and quercetin glucoside uptake from shallot and apple homogenates.

**Results:**

The in vitro digestion alone significantly decreased quercetin aglycone recovery from the shallot digestate (*p *< 0.05), but had no significant effect on quercetin-3-glucoside recovery (*p *> 0.05). Digestion increased the Caco-2 cell uptake of shallot quercetin-4'-glucoside by 2-fold when compared to the non-digested shallot. Despite the loss of quercetin from the digested shallot, the bioavailability of quercetin aglycone to the Caco-2 cells was the same in both the digested and non-digested shallot. Treatment with lactase increased quercetin recovery from the shallot digestate nearly 10-fold and decreased quercetin-4'-glucoside recovery by more than 100-fold (*p *< 0.05), but had no effect on quercetin recovery from apple digestates. Lactase treatment also increased shallot quercetin bioavailability to the Caco-2 cells approximately 14-fold, and decreased shallot quercetin-4'-glucoside bioavailability 23-fold (*p *< 0.05). These Caco-2 cells had lactase activity similar to that expressed by a lactose intolerant human.

**Conclusions:**

The increase in quercetin uptake following treatment with lactase suggests that dietary supplementation with lactase may increase quercetin bioavailability in lactose intolerant humans. Combining the digestion, the lactase treatment and the Caco-2 cell culture model may provide a reliable in vitro model for examining flavonoid glucoside bioavailability from foods.

## Background

Cardiovascular disease and cancer are the two most common causes of death in the United States and most industrialized nations. A diet high in fruits and vegetables has been correlated with a reduced risk for both cancer and heart disease [1,2]. It is thought that the phytochemicals found in fruits and vegetables may be responsible in part for these health benefits [3]. Phytochemicals from fruits and vegetables may inhibit cell proliferation, protect against oxidative stress, influence cell-signaling pathways, and reduce inflammation. Because these compounds appear to have such beneficial effects, interest has been raised in examining the bioavailability of these compounds.

A phytochemical of particular interest is quercetin, a strong antioxidant that is widely consumed in many fruits and vegetables. Quercetin has potential protective effects against both cancer and heart disease. Briefly, quercetin has been found to down regulate expression of mutant p53 in breast cancer cells, arrest human leukemic T-cells in G1, inhibit tyrosine kinase, and inhibit heat shock proteins [4]. Quercetin has been shown to decrease lipid peroxidation, inhibit cell proliferation, induce apoptosis, and inhibit platelet aggregation [5-8]. Because quercetin exhibits such a wide array of positive health effects, it is especially important to understand quercetin bioavailability from whole foods. Two widely consumed, good food sources of quercetin are apples and onions [9-13]. In most foods, quercetin does not exist in the aglycone form, but is, instead, conjugated. The type of sugar moiety to which quercetin is bound affects quercetin bioavailability. For example, quercetin in the apple is bound mainly to galactosides, rhamnosides, and arabinosides, and these quercetin conjugates are not well absorbed by the small intestine. The onion contains mainly quercetin glucosides, which are well absorbed by the small intestine [14].

More work is needed to understand the bioavailability of quercetin and other flavonoids from foods. The Caco-2 cell culture model is a well-established in vitro technique, extensively used to study intestinal cell absorption of compounds such as pharmaceuticals and nutrients; it is an excellent in vitro tool to study bioavailability of specific compounds. We have previously used the Caco-2 cell culture model to examine the uptake of quercetin from apple and onion extracts [15]. Using this model, we found that absorbed quercetin from onion extracts was significantly greater than from apple extracts, as expected [15]. Others have used the Caco-2 cell culture model to evaluate cell transport and/or accumulation of pure phytochemicals such as quercetin, quercetin glucosides, chrysin, flavone, epicatechin, proanthocyanidin, and carotenoids [16-23].

To further understand quercetin bioavailability, it is important to examine the effects of digestion on foods prior to intestinal uptake. An *in vitro *digestion has been paired successfully with the Caco-2 cell culture model to study iron and carotenoid bioavailability [23,24]. Use of the *in vitro *digestion with the Caco-2 cell culture model could be quite useful in more specifically analyzing quercetin bioavailability from foods. At this time there is little information available describing the effects of digestion on flavonoids from foods. Vallejo et al. [25] found that over 80% of total flavonoids were lost during an in vitro digestion of broccoli. In a study of ileostomy patients, Walle et al. estimated that the intestine might absorb 65–81% of major forms of dietary flavonoids after enzymatic hydrolysis [26].

A good *in vitro *model would aid in evaluating bioavailability of phytochemicals from foods by offering a simple method to screen for factors that may affect intestinal absorption of quercetin and quercetin glucosides, such as the food matrix, food processing, digestion, and interactions with other foods. Human and animal models can be expensive and time consuming, while a cell culture model allows for rapid, inexpensive screenings. The Caco-2 model has the potential to be a good model to measure quercetin absorption, however there are some drawbacks. Caco-2 cells have been shown to express significantly less lactase phlorizin hydrolase (LPH) than the average human small intestine [27]. Since this enzyme is most likely responsible for the first step in the metabolism of quercetin glucosides [28], this deficiency would clearly limit the ability of the Caco-2 cells to metabolize and absorb quercetin from quercetin glucosides.

Caco-2 cells used in our lab have expressed greater LPH activity (3 mU/mg protein) than other Caco-2 cells (0.3 mU/mg protein) [15], resulting in lactase activity similar to that expressed by enterocytes from a lactose intolerant human (2–10 mU/mg protein)[27]. The compound forskolin induced lactase phlorizin hydrolase activity four-fold in Caco-2 cells [29]. In weanling rats, lactose consumption increased lactase activity in the jejunum by three-fold [30]. Thus, we hypothesized that treating Caco-2 cells with either forskolin or lactase may raise lactase expression to rates comparable to humans, making the Caco-2 cell model a more valid model for screening quercetin glucoside bioavailability from food. If lactase activity cannot be induced in Caco-2 cells, treating food samples with lactase following the digestion procedure and prior to cell bioavailability assays may give more comparable results to humans.

The objectives of this study were (1) to develop an optimized in vitro digestion method for examining quercetin and quercetin glucoside recovery from digestates using onions and apples; (2) to examine the effect of lactase on shallot digestates; and (3) to examine Caco-2 cellular uptake of quercetin and quercetin glucosides from digested and lactase treated shallot.

## Methods

### Chemicals and materials

Shallots and onions (Northern Yellow) were obtained from a local grocery store. Apples (Red Delicious and Cortland varieties) were obtained from the Cornell Orchards (Cornell University, Ithaca, NY). Porcine pepsin, bile extract, pancreatin, lactase (beta-galactosidase, from *Kluyveromyces lactis*, activity of 3000 units/mL), quercetin, and quercetin-4'-glucoside were purchased from Sigma Chemical Company (St. Louis, MO). Quercetin-3-glucoside was purchased from Indofine Chemical Company, Inc (Hillsborough, NJ). Caco-2 cells were obtained from the American Type Culture Collection (Rockville, MD) and were cultured in Dulbecco's Modified Eagle Medium (DMEM; Gibco Life Technologies, Grand Island, NY) supplemented with 5% fetal bovine serum (Gibco Life Technologies, NY), 10 mM HEPES, 50 units/mL penicillin, 50 μg/mL streptomycin, and 100 μg/mL gentamicin, and were maintained at 37°C in 5% CO_2_.

### In vitro digestion

Two hundred grams of each food sample were chopped, blended for 5 min with 200 mL saline (140 mM NaCl, 5 mM KCl) using a Waring blender, and then homogenized using a Virtis 45 homogenizer. The total homogenates were aliquotted in 15 mL centrifuge tubes and stored at -20°C until use.

For the digestion treatment, 2 g aliquots of the food sample were placed in a centrifuge tube with an equal amount of saline. The pH was decreased to 2.0 by drop-wise addition of 1M HCl, and porcine pepsin was added to a final concentration of 1.3 mg/mL. The digestate was incubated in a shaking water bath at 37°C for 30 minutes. The pH of the digestate was then increased to 5.8 with the drop-wise addition of 1M NaHCO_3_. Porcine bile extract and pancreatin were added to a final concentration of 1.1 and 0.175 mg/mL, respectively. The pH was increased to 6.5 by drop-wise addition of 1M NaHCO_3_, and the samples were incubated for 1 hour in a water bath at 37°C. Following digestion the pH was decreased to 2 by addition of HCl and the digestates were stored at -80°C for further analysis.

To examine and optimize the effects of digestion time and pH on the recovery of compounds from the digestate, the above parameters were varied. To examine the effects of pepsin digestion time, the pepsin digestates were incubated for 0, 30, 60 or 90 minutes and then incubated with the intestinal digestion enzymes for 60 minutes. To examine the effects of intestinal digestion, the samples were incubated with pepsin for 30 minutes, then incubated with pancreatin and bile for 0, 30, 60, or 90 minutes.

The effects of 100 μM ascorbic acid and a nitrogen environment on quercetin and quercetin glucoside recovery from onion and apple digestates were examined. Following homogenization and prior to digestion, the food samples were mixed 1:1 with saline containing 200 μM ascorbic acid, leaving a final sample concentration of 100 μM ascorbic acid. During the digestion procedure described above, the samples were flushed constantly with nitrogen.

The effect of pH of either 6.5 or 7.0 during intestinal digestion on quercetin and quercetin-3-glucoside recovery was compared. The effect of the storage pH was examined by comparing digested samples having either a final pH of 2.0 or 6.5. The effect of storage pH on 20 μM pure quercetin and 20 μM quercetin-3-glucoside was examined by comparing recoveries from samples stored at pH = 2.0, 3.5, 5.0 or 7.0. All samples were stored overnight at -80°C. Prior to HPLC analysis samples were thawed and extracted 4 times with acidified ethyl acetate (pH 2.0), evaporated to dryness and reconstituted in 2 mL acidified methanol (pH 2.0).

### Lactase digestion

Doses of lactase (0.5 units to 3000 units per gram sample) were applied to 1 gram shallot extract and incubated for 15 minutes at 37°C. The final pH was brought to 2.0 and the samples were stored at -80°C. The time kinetics were examined by incubating 1 gram shallot extract with 100 units of lactase for 0, 15, 30, 60, 90, 120, 240, and 720 minutes.

The effect of both lactase and digestion were examined by digesting the samples with pepsin and pancreatin as described above, then incubating the samples with 100 units of lactase for 30 minutes at 37°C. Samples were stored overnight at -80°C.

Prior to HPLC analysis, all digestate samples were thawed and extracted 4 times with acidified ethyl acetate (pH 2.0), evaporated to dryness and reconstituted in 2 mL methanol.

### Uptake of quercetin-4'-glucoside and quercetin from shallot digestates by Caco-2 cells

Caco-2 cells were seeded at a density of 5 × 10^5 ^cells per well in a collagen coated 6-well, flat bottom plate and incubated at 37°C in a 5% CO_2 _environment. Caco-2 cells were used between passages #10–25, and the cells reached confluence approximately 5 days post seeding. Culture media was changed three times a week. On day 14 post seeding, the DMEM was removed and the cells were rinsed three times with phosphate buffered saline (PBS).

Shallot homogenates were digested as previously described and were placed directly on the 14 day old Caco-2 cells, or the samples were diluted 1:2 or 1:4 in HBSS (Hank's Balanced Salt Solution). Cells were also incubated with non-digested shallot homogenates for comparison. For each treatment, two wells of cells were used for each sample, and each treatment was repeated in triplicate.

To examine the effect of lactase on quercetin uptake from shallots, shallots were digested then incubated with lactase (50, 100, 300, and 1000 units/g shallot) for 20 minutes at 37°C. The digested shallot homogenates and the digested plus lactase treated shallot homogenates were diluted 1:2 in HBSS and placed on the cells. In all experiments, Caco-2 cells were incubated with treatment for 30 minutes at 37°C in 5% CO_2_. The shallot treatment and HBSS was removed and the cells were rinsed three times with 20% methanol in PBS. Cells were scraped in acidified methanol (pH = 2.0) and the wells were rinsed three times with methanol. The scraped cells were sonicated for 15 minutes, centrifuged at 1600 g for 5 minutes, and the methanol supernatant was collected. The cells were rinsed three more times with methanol, the supernatants were collected and the methanol extracts were evaporated to dryness under nitrogen and reconstituted in 400 μl acidified methanol for HPLC analysis.

### Induction of lactase activity in Caco-2 cells

Caco-2 cells were seeded at a density of 5 × 10^5 ^cells per well in a 6-well flat bottom plate. The cells were cultured in DMEM spiked with different doses of either lactose (10, 50, 100, 500, and 1000 μM), or forskolin (1, 10, 50, 100, and 200 μM). Media was changed every two days, and cells were harvested and lactase activity was measured at 14 days post-seeding. Lactase activity of Caco-2 cells was measured using a method adapted from Dahlqvist [31]. Cells were trypsinized, collected, centrifuged and resuspended in homogenization buffer (50 mM sodium phosphate; 1 mM EDTA; 10 mM dithiothreitol; protease inhibitor cocktail, Sigma Chemical Co., St. Louis, MO). Cells were homogenized 5 times for 30 seconds with 1 minute of cooling between bursts using a benchtop homogenizer. Homogenates were treated with 56 mM lactose and incubated at 37°C for 60 minutes. Glucose oxidase, peroxidase, and o-dianisidine were applied to the cell homogenates and the final colored products were measured at 420 nm using a spectrophotometer [31]. The results were compared to a glucose standard curve to determine the amount of glucose released by lactase in the Caco-2 cell monolayer. Protein was determined from crude cell homogenates colorimetrically using the Lowry method with comparisons to a bovine serum albumin standard curve. Results are expressed as milliunits/mg of protein, and one unit is defined as the lactase activity that hydrolyzes 1 μmole of lactose per minute at 37°C.

### HPLC analysis

Quercetin and quercetin-3-glucoside content of untreated homogenates, digestates, and Caco-2 cell extracts were determined using an RP-HPLC procedure with a Supelcosil LC-18-DB column (150 mm × 4.6 mm, and 3 μm pore size). Waters 515 HPLC pumps (Waters Corp., Milford, MA) and a Waters 2487 dual wavelength absorbance detector (Waters Corp., Milford, MA) set at 370 nm were used for all HPLC analysis. Quercetin, quercetin-3-glucoside, and quercetin-4'-glucoside were used as standards. For the analysis of quercetin, quercetin-3-glucoside, and quercetin-4'-glucoside in the apple peel extracts, shallot extracts, and digestate extracts, the solvent system used was (A) acidified water (pH 2.0; triflouroacetic acid) and (B) acetonitrile. The gradient method was the following: 0.0 min, flow rate = 1.4, (A) 90% and (B) 10%; 53 min, flow rate = 1.5, (A) 80% and (B) 20%; 58 min, flow rate = 1.7, (A) 65% and (B) 35%; 64 min, flow rate = 1.4, (A) 90% and (B) 10%. Twenty μL injections were made for each sample. Quercetin, quercetin-3-glucoside, and quercetin-4'-glucoside concentrations in the apple peel extracts, shallot extracts, and in the digestates were extrapolated from the pure quercetin and quercetin-3-glucoside standard curves.

### Statistical analysis

All data were reported as means ± SD for three replicates of each treatment. An analysis of variance (ANOVA) was used to compare results between treatment groups, and pairwise multiple comparisons were performed using Fisher's LSD with an individual error rate of 0.05. The statistical analysis was completed using Minitab Release 12 software (State College, PA).

## Results

### In vitro gastrointestinal digestion

The total pepsin digestion time and pancreatin/bile digestion time had little to no effect on recovery of both quercetin and quercetin-3-glucoside from apple and onion homogenates when treated for up to 60 minutes (data not shown). After 90 minutes of pepsin digestion and 90 minutes of pancreatic digestion, quercetin and quercetin 3-glucoside in both the apple and the onion decreased slightly. Based on these results we chose to use a 30-minute pepsin digestion and a 60-minute pancreatin/bile digestion. In past studies, quercetin from onion and quercetin from quercetin-4-glucoside supplements reached the plasma in less than an hour following consumption by human volunteers [32]. Therefore, long in vitro digestion times were not necessary to mimic human digestion of quercetin compounds from apples and onions.

The presence of ascorbic acid and nitrogen had no effect on quercetin or quercetin-3-glucoside recoveries from the digestates (data not shown). Quercetin and quercetin-3-glucoside recoveries from digested samples treated with ascorbic acid, nitrogen or both ascorbic acid and nitrogen were not different from the recoveries from the untreated digested samples.

**Figure 1:**
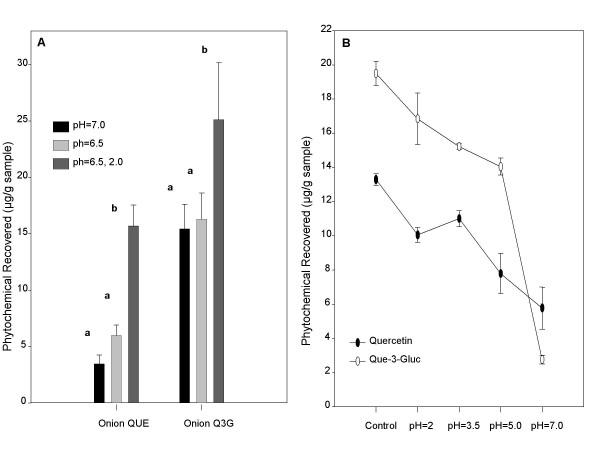
The effects of intestinal digestion pH and acidic storage on quercetin and quercetin-3-glucoside recovered from digested onions (A) and the effects of storage pH on digested pure quercetin and quercetin-3-glucoside (B). Samples were digested for 30 minutes with pepsin, 60 minutes with pancreatin and bile, and then stored at -80°C. Each point represents the mean ± standard deviation of triplicate observations within the same experiment. Different letters indicate significantly different observations within each compound (*p *< 0.05).

The factor that had the greatest effect on recovery was pH (Figure 1A). Quercetin is less stable at higher pH, therefore the effect of pH during intestinal digestion was examined. Recoveries of quercetin and quercetin-3-glucoside after intestinal digestion at pH 7.0, when compared to pH 6.5, were not significantly different (*p *> 0.05). However, following overnight storage at -80°C, the samples stored at pH 2.0 had significantly greater quercetin and quercetin-3-glucoside recoveries than samples stored at pH 6.5 and 7.0 (*p *< 0.05). Pure quercetin and quercetin-3-glucoside were also more stable at lower storage pH following digestion (Figure 1B). At pH 2.0, the recoveries for pure quercetin and quercetin-3-glucoside were 74.8% and 86.2% when compared to the control. At the highest pH (7.0), recoveries for quercetin and quercetin-3-glucoside were 46.5% and 13.9%, respectively.

**Figure 2:**
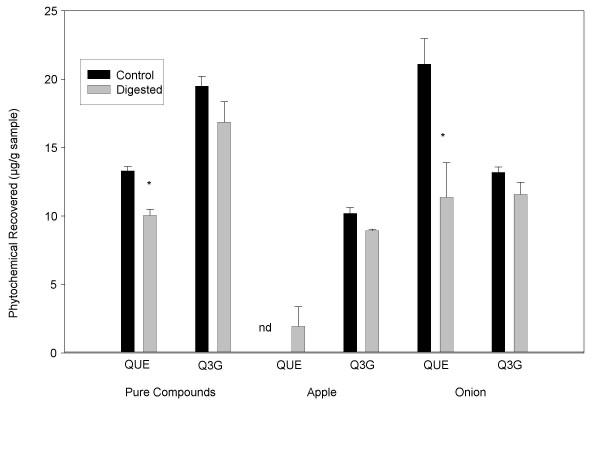
The effects of digestion on pure quercetin and quercetin-3-glucoside and quercetin and quercetin-3-glucoside from apple and onion. Each point represents the mean ± standard deviation of triplicate observations within the same experiment. An asterisk indicates a significant difference between the control and the digestate (*p *< 0.05).

Based on these results, optimal digestion conditions were decided to be: pepsin digestion at pH 2.0 for 30 minutes, pancreatin/bile digestion at pH 6.5 for 60 minutes, and a final storage at pH 2.0. Ascorbic acid and nitrogen treatments were not continued. Using these conditions, the effect of digestion on quercetin and quercetin-3-glucoside recoveries from apples, onions, and pure quercetin and quercetin-3-glucoside was examined (Figure 2). Following digestion, recoveries of pure quercetin-3-glucoside, apple quercetin-3-glucoside, and onion quercetin-3-glucoside were similar to recoveries from non-digested samples (*p *> 0.05). Quercetin-3-glucoside recoveries from the digested apple, onion, and pure compound were 87.7, 89.5, and 86.4%, respectively. Quercetin recovery was significantly reduced in digested pure quercetin and digested onion samples, when compared to non-digested samples (*p *< 0.05). Quercetin recovery was lower than quercetin-3-glucoside recovery and tended to vary more, depending on the food matrix: 52.5% and 74.3%, from the onion and pure compound, respectively. There was no significant difference in quercetin recovery between to the non-digested and digested apple homogenates. There was only trace or no quercetin in non-digested apple homogenates, so the appearance of any quercetin following digestion resulted in a net increase.

### Uptake of quercetin-4'-glucoside and quercetin from shallot digestates by Caco-2 cells

**Figure 3:**
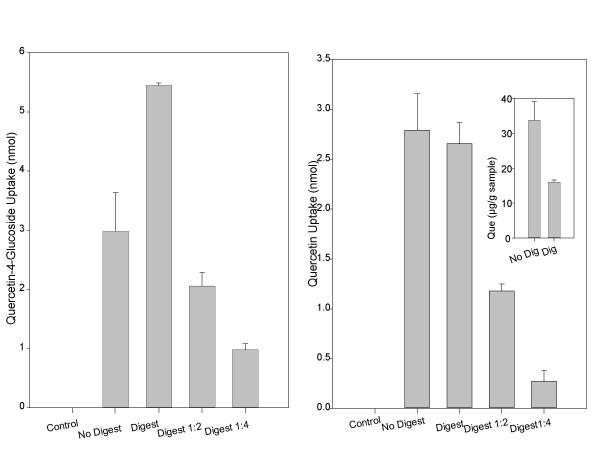
Caco-2 uptake of quercetin-4-glucoside (A) and quercetin (B) from digested and non-digested shallot homogenates. Shallot homogenates were digested for 30 minutes with pepsin at pH 2.0 and for 60 minutes with pancreatin/bile at pH 6.5. Digestates were directly placed on cells or diluted 1:2 or 1:4 in HBSS and then placed on cells. Cells were incubated with digestates for 30 minutes at 37°C. The imbedded graph in (B) shows quercetin recovery from shallots following the digestion procedure only. Each bar represents the mean ± standard deviation of triplicate observations within the same experiment. Different letters indicate significantly different observations within each compound (*p *< 0.05).

Quercetin-4'-glucoside and quercetin were absorbed by Caco-2 cells following treatment with both digested shallot and non-digested shallot homogenates (Figure 3). Quercetin-3-glucoside was not detected in any sample. Quercetin-4'-glucoside uptake by the Caco-2 cells increased by approximately 2-fold following digestion (*p *< 0.05). Caco-2 cells treated with shallot homogenate absorbed approximately 2.9 ± 0.65 nmol of quercetin-4-glucoside, and Caco-2 cells treated with digested shallot absorbed 5.4 ± 0.04 nmol. Quercetin aglycone recovery from the digested shallot extract was only 47% that of the non-digested homogenate (Figure 3B insert), however quercetin uptake from the digested samples was similar to the non-digested samples (*p *> 0.05). Caco-2 cells absorbed 2.8 ± 0.4 nmol and 2.7 ± 0.2 nmol quercetin from the non-digested and digested shallot homogenates, respectively. Absorption of both quercetin-4-glucoside and quercetin from digested shallot followed a dose response. The Caco-2 cells absorbed quercetin 4'-glucoside and quercetin incrementally less from the digested samples that were diluted 1:2 or 1:4 in HBSS.

### Induction of lactase activity in Caco-2 cells

Addition of lactose and forskolin, a specific inducer of lactase [29], to Caco-2 cells did not significantly increase lactase activity of Caco-2 cells. The lactase activity of all cells ranged from 2–4 mU/mg protein in all treatments.

### Lactase Digestion

**Figure 4:**
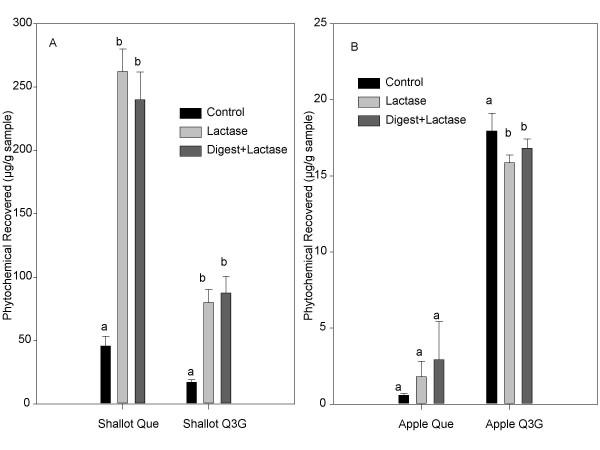
The effects of lactase and a combined lactase and digestion treatment on quercetin and quercetin-3-glucoside recovery from shallot (A) and apple (B) homogenates. Each point represents the mean ± standard deviation of triplicate observations within the same experiment. Different letters indicate significantly different observations within each compound (*p *< 0.05).

Treatment with 100 units lactase/g sample had a significant effect on both quercetin and quercetin-3-glucoside recoveries from the shallot (*p *< 0.05; Figure 4). Quercetin recovery from shallot digestates increased 5.5 fold, from 47.5 ± 7.6 μg/g sample in the untreated digestate to 262.2 ± 17.6 μg/g sample in the lactase treated sample. The lactase plus digestion treatment resulted in a non-significant decrease in quercetin recovery compared to the lactase only treated samples. Quercetin-3-glucoside from shallot digestates also increased approximately 5 fold, from 17.3 ± 1.7 μg/g sample to 80.0 ± 10.3 μg/g sample following the lactase treatment. The effect of lactase on the apple samples was not as great. Quercetin-3-glucoside recovery decreased slightly, while changes in quercetin levels were not significant (*p *> 0.05).

**Figure 5:**
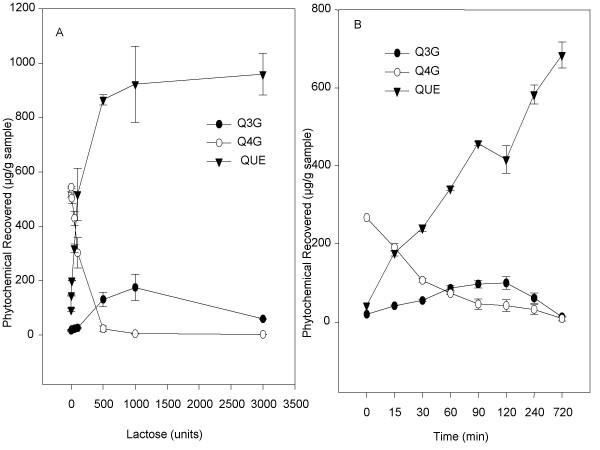
Dose response (A) and kinetics (B) of lactase on quercetin and quercetin glucoside recovery from shallots. Homogenized shallots were incubated 60 for minutes with 10, 50, 100, 300, 500, 1000, or 3000 units of lactase/mL sample. Homogenized shallots were incubated with 100 units lactase/mL sample for 15, 30, 60, 90, 120, 240, and 720 minutes. Each point represents the mean ± standard deviation of triplicate observations within the same experiment.

Because treating shallots with lactase increased quercetin recovery so greatly without significantly decreasing quercetin-3-glucoside recovery, the effect of lactase on quercetin-4'-glucoside recovery was also examined. More quercetin-4'-glucoside is found in shallots when compared to quercetin-3-glucoside. As the dose of lactase increased, quercetin-3-glucoside recovery increased from 18.2 ± 3.7 up to 175.5 ± 48.1 μg/g shallot at the 1000 unit dose and then decreased to 60.2 ± 2.0 μg/g at the 3000 unit dose (Figure 5A). As the dose of lactase increased, quercetin recovery increased and quercetin-4'-glucoside decreased (Figure 5A). The increase in quercetin was quite dramatic. Recovery of quercetin from untreated shallot samples was 93.7 ± 2.2 μg quercetin per gram sample, and at the highest lactase dose, recovery of quercetin from shallot samples was 958.8 ± 76.1 μg quercetin per gram shallot. Quercetin-4'-glucoside recovery decreased from 518.2 ± 10.7 μg/g shallot from the untreated sample to 3.2 ± 0.8 μg/g shallot at the highest treatment dose. A similar trend was seen for all compounds in the kinetic experiment. As the incubation time increased, recoveries of quercetin increased and quercetin-4'-glucoside decreased (Figure 5B). Quercetin-3-glucoside increased through two hours, and then decreased following four and eight hours of incubation with lactase. In both the dose response and kinetic experiments, increases in quercetin recoveries were greater than decreases in quercetin-3-glucoside or quercetin-4'-glucoside recoveries.

### Caco-2 cell uptake of quercetin and quercetin glucosides following lactase treatment

**Figure 6:**
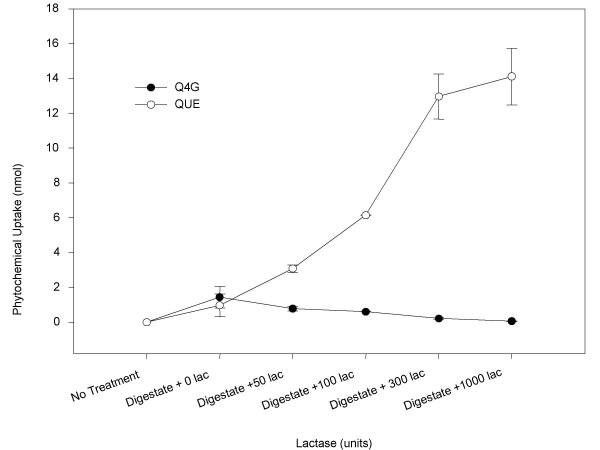
Caco-2 uptake of quercetin-4-glucoside and quercetin from digested shallot and digested plus lactase treated shallot. Shallot homogenates were digested for 30 minutes with pepsin at pH = 2.0 and for 60 minutes with pancreatin/bile at pH = 6.5. Digested shallots were then treated with either 50, 100, 300, or 1000 units lactase/mL sample for 20 minutes. Samples were diluted 1:2 and then placed on the cells for 30 minutes. Each point represents the mean ± standard deviation of triplicate observations within the same experiment.

The addition of lactase following the pepsin and pancreatin/bile digestion significantly increased the amount of quercetin absorbed by the Caco-2 cells with a significant decrease in the amount of quercetin-4'-glucoside absorbed by the Caco-2 cells (*p *< 0.05, Figure 6). Quercetin uptake increased from 0.98 ± 0.67 nmol from the digested sample up to 14.1 ± 1.6 nmol from the digested plus 1000 units lactase treated sample. Quercetin-4'-glucoside uptake by Caco-2 cells decreased as the dose of lactase increased, however the increase in quercetin was more dramatic than the decrease in quercetin-4'-glucoside. Quercetin-3-glucoside uptake was not detected.

## Discussion

### In vitro digestion

Previously, our laboratory determined that our Caco-2 cells had the potential to be used as a model to study quercetin bioavailability from onions and apples [15]. In the present study, we modified an *in vitro *digestion procedure and combined it with the Caco-2 cell model to give a more comprehensive examination of quercetin and quercetin glucoside bioavailability in Caco-2 cells.

The digestion procedure modified in these experiments resulted in quercetin-3-glucoside recoveries that ranged from 78.9 to 89.5% and quercetin recoveries that ranged from 47.3 to 74.3%. In both cases, the lowest digestate recoveries were from the shallot. Interestingly, quercetin recovery from both the onion and shallot was considerably lower than from the pure compound. This difference is not yet explained, but could potentially be due to interactions with other compounds found in the onion and shallot.

In all cases, quercetin recovery from digestates was lower than quercetin-3-glucoside. The glucoside moiety may lend stability to quercetin-3-glucoside during digestion, and could contribute to its greater bioavailability *in vivo *as well. Quercetin aglycone may be more susceptible to oxidation or other degradation during exposure to both the digestive enzymes and the variations in pH in the stomach and intestine. Ascorbic acid and nitrogen, both added to help decrease oxidation, had no effect on quercetin or quercetin-3-glucoside recoveries from digested onions.

The factor that had the greatest effect on both quercetin and quercetin-3-glucoside stability in the digestate was pH. Recoveries of digested pure compounds and digested compounds from the onion homogenates were significantly less if stored at a pH of 6.5 or 7.0 than if stored at pH 2.0 at -80°C (Figure 1). Vallejo et. al [25] used an *in vitro *digestion method to measure the effect of digestion on a variety of compounds from broccoli. Following the pepsin and pancreatic digestion, they recovered only 16% of total flavonoids, and the main flavonoids found in the broccoli were quercetin and kaempferol glycosides. The recovery of quercetin and quercetin-3-glucosides, flavonoids common to the foods in our study, was much higher than 16% from the digested shallot, onion and apple. It has been estimated that quercetin glucoside bioavailability may be as high as 80% in humans and quercetin bioavailability may range from 35–53% [26,33]. Our results would appear to be more reasonable estimates, if indeed, quercetin and quercetin glucoside bioavailability lies within the approximated ranges found by Walle et. al [26,33].

### Effect of digestion on quercetin and quercetin glucoside uptake by Caco-2 cells

Digestion of the shallot resulted in decreased recoveries of both quercetin and quercetin-3-glucoside, therefore it was expected that digestion might decrease the bioavailability of these compounds as well. Following digestion, quercetin aglycone in the shallot was decreased by approximately 50%; however, quercetin bioavailability was unchanged following digestion compared to the non-digested samples. This means that the digestion procedure must degrade quercetin, and simultaneously enhances the bioavailability of quercetin, bringing the cellular uptake back to comparable levels with the non-digested samples. Quercetin-4'-glucoside uptake by the Caco-2 cells from the shallot increased by approximately 2-fold following the in vitro digestion. We hypothesize that the digestion procedure may have released more compounds from the food matrix leaving them more available for uptake by the Caco-2 cells. The digestion procedure may also have improved solubility of the compounds, increasing their absorption by the Caco-2 cells.

Quercetin-3-glucoside was not detected in the Caco-2 cells following treatment with shallot. Quercetin-3-glucoside is a minor compound in the shallot, and it is believed that the levels were below our detection limit. In the past, we found that Caco-2 cells did absorb trace amounts of quercetin-3-glucoside from shallot extracts [15]. In the previous experiments, shallots were first extracted with ethanol and ethyl acetate, and finally reconstituted and concentrated in methanol. This procedure produced more concentrated shallot extracts for cell treatment than with our current procedure. In the current study, shallot homogenates and digestates were too dilute to detect small changes in initial concentrations or in cellular uptake of quercetin-3-glucoside, which was below the detection limit.

Strong evidence suggests that quercetin glucosides are more bioavailable in humans than the quercetin aglycone, however it has not yet been determined why this is the case. Based on the digestion data, quercetin glucoside is more stable following the *in vitro *digestion conditions than quercetin and is therefore more likely to reach the intestine intact. Quercetin-4'-glucoside bioavailability in Caco-2 cells was increased nearly 2-fold following digestion and quercetin absorption was not changed (Figure 3). It has been hypothesized that absorbed intact quercetin glucosides are then quickly hydrolyzed by cytosolic β-glucosidase to quercetin aglycone [34]. It has also been hypothesized that the major pathway for quercetin glucoside absorption begins with hydrolysis by LPH. Deglycosylation of quercetin glucosides at the brush border membrane positions the resulting aglycone in a prime position for diffusion across the brush border. The deglycosylation of the quercetin glucoside would result in a higher concentration of aglycone at the apical enterocyte membrane and potentially increase the rate of absorption [35].

### Effect of lactase on quercetin and quercetin-4'-glucoside uptake by Caco-2 cells

**Figure 7:**
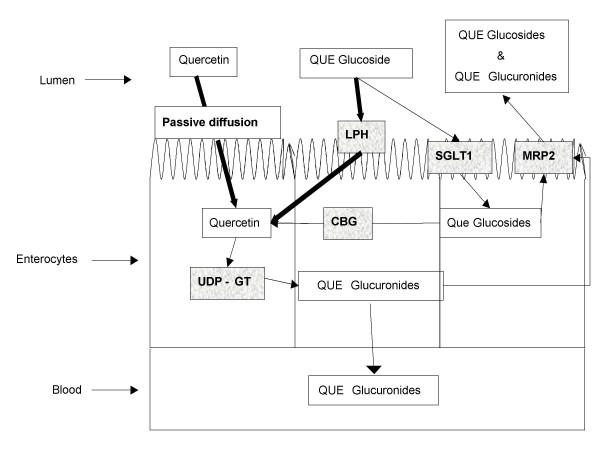
Potential mechanism of quercetin and quercetin glucosides uptake by enterocytes. LPH, lactase phlorizin hydrolase; SGLT1, sodium-dependent glucose transporter 1; CBG, cytosolic B-glucosidase; MRP2, multi-drug resitance protein 2; UDP-GT, UDP glucuronosyl transferase; QUE, quercetin.

The potential pathways for quercetin glucoside and quercetin metabolism and absorption can be seen in Figure 7. Quercetin aglycone passively diffuses across the apical membrane and is then glucuronidated. Evidence strongly suggests that quercetin glucosides are first hydrolyzed by the lactase site of lactase phlorizin hydrolase prior to diffusion across the apical membrane [36]. Quercetin glucosides may also be transported into the cell by the sodium-dependent glucose transporter1 (SGLT1) and then hydrolyzed by the cytosolic beta-glucosidase. Quercetin-3-glucoside is not a good substrate for cytosolic beta-glucosidase [37]. Since research has shown that both quercetin-3-glucoside and quercetin-4'-glucoside are similarly bioavailable in humans [14,38], this could be an indication that hydrolysis by LPH and the subsequent passive diffusion of quercetin into the cell is the main pathway for quercetin glucoside absorption across the brush border. Following hydrolysis and incorporation into the cells, quercetin aglycone is then glucuronidated. Quercetin glucosides and possibly quercetin glucuronides are then transported back into the lumen by multidrug resistance protein 2 (MRP2). Conjugated quercetin metabolites also eventually reach circulation, but the transporter involved in transporting them across the basolateral side is still unknown.

Since lactase is an important enzyme in the metabolism and subsequent absorption of quercetin glucosides, lactose intolerant individuals may have a reduced capability to hydrolyze quercetin glucoside for further absorption across the small intestinal wall. Many lactose intolerant people use commercial lactase to break down lactose. Not only might this enzyme help improve digestibility of lactose, but it may also increase bioavailability of quercetin from foods. Initial lactase treatments were applied both to shallot and apple homogenates and digestates. Lactase had little to no effect on apple samples. Apples contain quercetin bound mainly to galactosides, rhamnosides, and xylosides, conjugates that would not be readily hydrolyzed by lactase. However, lactase treatment had great effects on shallot and onion digestates. The shallot and onion are high in quercetin glucosides, mainly quercetin-4'-glucoside, quercetin-3-glucoside, and quercetin-3,4'-diglucoside, compounds readily hydrolyzed by lactase. Treatment with lactase in the range of 15 units/mg sample up to 1000 units/mg sample, significantly increased both quercetin and quercetin-3-glucoside recovery in shallot homogenates and digestates. The increase in quercetin-3-glucoside is most likely a result of deglycosylation of quercetin-3,4'-diglucoside. Rhodes et al. [39] found that over time quercetin-3,4'-diglucoside in chopped onion will autolyze to monoglucosides, and within 24 hours the diglucoside will completely disappear. This may also explain the increase in quercetin-3-glucoside over time. Quercetin-4'-glucoside decreased following treatment with lactase as expected. Results from this work suggest that quercetin-4'-glucoside is utilized by lactase prior to quercetin-3-glucoside. Interestingly, the increase in total quercetin was greater than the decrease in quercetin-4'-glucoside. Digestion with lactase may release quercetin from the food matrix as well, making it more available for absorption.

Following hydrolysis of quercetin glycosides, it has been hypothesized that quercetin is quickly glucuronidated, and quercetin glucuronides are then found circulating in the plasma [40]. In the current studies, these Caco-2 cells showed no signs of glucuronidating quercetin following quercetin absorption, but do express some LPH activity as was evident by the increased quercetin uptake from shallots and from the lactase activity assays [15]. Thus, these Caco-2 cells have the potential to be a good model of quercetin absorption, but not of further metabolism.

A good model of quercetin glucoside bioavailability should incorporate lactase activity. The Caco-2 cells used for these experiments expressed lactase activity similar to that of a lactose intolerant human (2–10 mU/mg protein). These cells had approximate lactase activity of between 2–4 mU/mg protein, consistent with what we previously reported [15]. Lactose tolerant humans tend to have intestinal lactase activity that ranges from 20–80 mU/mg protein [41]. Forskolin and lactose did not induce lactase activity in our Caco-2 cells. The lactase activity of our cells was nearly 10 times higher than previously reported values for Caco-2 cells of 0.3 mU/mg protein of lactase activity [27], and it is quite possible that the lactase enzyme in our Caco-2 cells is already expressed to the fullest extent.

**Figure 8:**
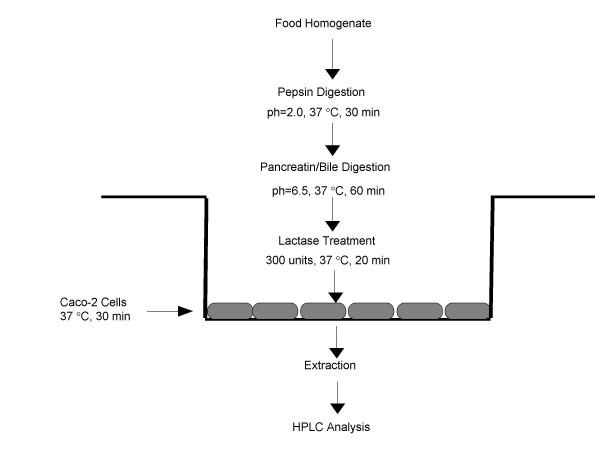
Caco-2 cell culture model for examining quercetin bioavailability from foods.

Since lactase activity could not be increased in the Caco-2 cells, we combined a lactase treatment with digestion to provide an intestinal uptake model that is more comparable to a lactose tolerant human, or a lactose intolerant human ingesting a lactase digestive aid to help improve lactose digestion. Not only did lactase increase the amount of quercetin in digested shallot homogenates, but it also increased the amount of quercetin taken up by the Caco-2 cells from the digested shallot extracts. This suggests that a lactase containing digestive aid may increase absorption of quercetin from onions in lactose intolerant humans. Combining the Caco-2 cell model with an in vitro digestion, simulating stomach and small intestinal digestion, and a lactase digestion may provide a more useful model to examine and screen for bioavailability of flavonoid glucosides from common foods (Figure 8).

## Conclusions

Following an in vitro stomach and small intestinal digestion, the recovery of quercetin and quercetin-3-glucoside is optimized at a storage pH at or below 3.5. Storage at pH above 3.5 results in loss of most of the compounds. The in vitro digestion increased the uptake of shallot quercetin-4'-glucoside by the Caco-2 cells. Quercetin uptake by the Caco-2 cells was similar between digested and non-digested samples, despite the fact that approximately 50% of shallot quercetin is lost during digestion. Treating shallot digestates with lactase increased the recovery of quercetin aglycone 10-fold and decreased the recovery of quercetin-4'-glucoside. Lactase treatment increased the bioavailability of quercetin aglycone 14-fold and decreased the bioavailability of quercetin-4'-glucoside to the Caco-2 cells. Combining pepsin, pancreatin/bile, and lactase digestions with the Caco-2 cell culture monolayer may results in a useful model for studying flavonoid bioavailability from foods.

Many advances have been made in understanding flavonoid bioavailability, however many questions still remain unanswered. Food processing, interactions with other compounds as well as with other foods are all factors that may affect bioavailability of flavonoids. A simple and inexpensive screening model would be beneficial as a first step in examining many of these factors. The Caco-2 cell model has the potential to be a good model for analyzing intestinal uptake of quercetin, quercetin glucoside and other flavonoids. While the Caco-2 cell model will never be an exact replica of a human small intestine, it could provide a valuable tool for initial screenings of large quantities of samples relatively quickly and inexpensively. In this study, it was found that a simple digestive aid such as lactase might increase quercetin bioavailability. This may be of importance to lactose intolerant people. Such significant trends may be examined in more detail using human or animal models.
